# Newborn Sickle Cell Disease Screening Using Electrospray Tandem Mass Spectrometry

**DOI:** 10.3390/ijns4040035

**Published:** 2018-11-24

**Authors:** Yvonne Daniel, Charles Turner

**Affiliations:** 1 Viapath, Guy’s & St Thomas Hospital, London SE17EH, UK; 2 WellChild Laboratory, Evelina London Children’s Hospital, London SE17EH, UK

**Keywords:** screening, sickle cell disease, newborn, mass spectrometry

## Abstract

There is a growing demand for newborn sickle cell disease screening globally. Historically techniques have relied on the separation of intact haemoglobin tetramers using electrophoretic or liquid chromatography techniques. These techniques also identify haemoglobin variants of no clinical significance. Specific electrospray ionization-mass spectrometry-mass spectrometry techniques to analyse targeted peptides formed after digestion of the haemoglobin with trypsin were reported in 2005. Since this time the method has been further developed and adopted in several European countries. It is estimated that more than one million babies have been screened with no false-negative cases reported. This review reports on the current use of the technique and reviews the related publications.

## 1. Introduction

The first report of newborn screening for sickle cell disease (SCD) by mass spectrometry (MS) utilised matrix-assisted laser desorption-time of flight (MALDI-TOF) [1]. Intact haemoglobin chains were initially analysed followed by mass mapping of trypsin-active MS targets to localise the mutation site. This proof of concept had not translated into large-scale trials or prospective screening until recently. Newborn screening using MALDI-TOF is covered in a separate article in this issue and it will not be reviewed further here. The other ionisation mode for MS in common use is electrospray ionisation (ESI). ESI of undigested haemoglobin produces a large number of multiply charged ions, formed in the source when charged droplets are dried in the presence of an inert gas such as nitrogen. The ions formed can then be analysed.

Wild et al. [2] reported the use of ESI-MS to analyse intact globin chains in 147 newborn blood spots. The protocol utilised software to produce a deconvoluted profile spectrum displaying the α, β, and γ globin peaks along with peaks of variant haemoglobins with a mass shift sufficiently different from normal to allow resolution. This allowed detection of sickle globin and other variants with a mass difference of –30 daltons (Da) from normal β chains but did not clearly resolve those clinically significant variants with a mass difference of 1 Da. The method was not specific for Hb S, as there are 5 amino acid substitutions which result in a mass difference of 30 Da. The authors concluded that the technique allowed for detection of sickle cell disease, but samples found to have a positive signal for Hb S would require confirmation by an alternate technique. No large-scale trial of this ESI-MS approach has been published. In 2005, Daniel et al. [3] published the first report of a targeted ESI-MSMS approach, based on tryptic digestion. Developments of this method have been widely adopted into active newborn screening programmes and are the main subject of this review.

## 2. The MSMS (Tandem Mass Spectrometry) Technique

ESI coupled to tandem mass spectrometry (MSMS), in which ions formed are selected in a first quadrupole (Q1), product ions are produced via collision in Q2 and detected in Q3, is in common use in newborn screening for small molecules such as amino acids and acylcarnitiines. The 2005 paper of Daniel et al. [3] achieved targeted detection of haemoglobin variants including clinically significant variants with 1 Da mass difference from normal using ESI-MSMS with a series of experiments run simultaneously on whole blood samples digested with trypsin. Trypsin cleaves peptide bonds adjacent to arginine and lysine residues producing a predictable and reproduceable series of peptide fragments. As the clinically significant haemoglobin variants have been well characterised, the amino acid sequences are known along with the expected mass differences from normal. The target beta chain haemoglobin variants were Hb S and Hb C, Hb D^Punjab^ and Hb O^Arab^, and Hb E which occur in tryptic peptides (T) 1, 13, and 3 respectively. Selected reaction monitoring experiments were designed to select the required tryptic peptide in Q1 and a specific fragmentation ion in Q3 generated following collision induced fragmentation in Q2. Experiments were designed for wildtype and the designated haemoglobin variants. Unlike the previous method using intact chains, this method was targeted and highly specific. Additionally, the use of ESI-MSMS allowed rationalisation of equipment and better use of existing resources. The tryptic digestion had been simplified by the demonstration that informative peptides were produced within 30–45 min and that chemical denaturation of the protein or purification of haemoglobin prior to the addition of trypsin was unnecessary. A patent was awarded to the final method in 2006. The method of Daniel et al. [3] was commercialised by SpOtOn Clinical Diagnostics from 2013, and CE-marked reagent kits were made available in 2015. The kits include stable isotope-labeled Hb S peptide and trypsin, and peptide standards for instrument optimization are also available from the company. Dried blood spot samples are punched (3 mm spot), stable isotope and trypsin are added, and the samples incubated at 37 °C for 30 min. Following the addition of mobile phase to stop the reaction and act as diluent, samples are introduced into the MSMS using flow injection (without chromatography). The method utilizes simple acetonitrile/water/formic acid mobile phases as used for newborn screening for amino acids and acyl carnitines. The stable isotope-labeled Hb S allows sample-by-sample assurance of trypsin activity and MSMS performance.

## 3. Review of Published Results

The initial method was validated in a project funded by the NHS Sickle Cell & Thalassaemia Screening programme, which compared newborn blood spots analysed by the existing isoelectric focussing technique (IEF) in Leeds with the ESI-MSMS technique [4]. Over 40,000 blood spots were tested between August 2007 and August 2008. The results were analysed by reviewing the abundance ratio of the variant peptide to the corresponding wild-type peptide with no discrepant results observed, shown in Table 1.

Subsequently Boemer et al. [5] reported the results of 2082 newborn samples screened using a slightly modified MSMS method with the same principle. Results were compared with IEF and high performance liquid chromatography (HPLC). Only haemoglobins S, C, E, and wild-type β and γ were targeted with multiple experiments for each haemoglobin of interest. No discrepancies were reported. In 2011, the group reported a review of three years’ experience presenting the results of 43,736 newborn samples [6]. First-line MSMS analysis identified 444 samples as screen-positive. All were confirmed using molecular techniques. The paper also reported ranges obtained for the amalgamated ratio data of the variant to corresponding wild-type peptides as well as the β to γ peptides used to screen for β thalassaemia disease. These showed clear discrimination between unaffected and affected cases within each of the investigated disease categories, as well as carriers for the haemoglobin variants. The group noted the lack of an experiment to detect Hb Bart’s and therefore possible Hb H disease and noted the possibility that Hb S co-inherited with other clinically significant variants not targeted in the experimental protocol would have a result pattern identical to that of a sickle carrier.

Moat et al. [7] used the SpOtOn kit method in a study to inform the introduction of newborn screening for sickle cell disease in Wales. The published protocol built on the use of ratios and utilised locked software algorithms to screen for sickle cell and β thalassaemia disease, the latter as a clinically significant by-product. The only parameters available and routinely reviewed by the operator were those required to check for appropriate tryptic sample digestion, prematurity, and transfusion status. The developed algorithms prevented the identification of most sickle cell carriers and all carriers and homozygotes of C, D^Punjab^, O^Arab^, and E, as the operator was not alerted to the results of these experiments unless Hb S was detected at levels above the designated ratio action values. Ratio action values were set using the residual blood spots of 2154 normal and 675 known positive cases and were subsequently evaluated using 13,249 blood spots run in parallel with HPLC. The protocol identified some Hb S carriers as ratio action values were set sufficiently low to ensure that all possible cases of coinheritance of Hb S and β plus thalassaemia were detected. Unblinding of the data revealed a further 328 cases of infants who were carriers of either Hb S, C, D^Punjab^, or E, which had not been identified to the operator using the locked protocol. As the protocol is designed not to identify cases that are carriers of Hb S, it does not permit the detection of rare Hb variants that interact with Hb S. As these rare mutations are not targeted in the protocol, only the sickle mutation will be detected, giving screening results that mimic that of a sickle carrier. Examples include Hb Maputo and Hb North Shore.

An update and three-year review of the Welsh screening programme was published in 2017 [8]. At the time of writing, 100,456 babies had been screened using the protocol. Findings were similar to those of previous studies (10 true-positive sickle cell disease cases and 6 false positives) with no false negatives reported. The latter six cases were sickle cell carriers with results that fell above the action value in the Welsh Programme, which aims to detect only sickle cell disease. Such cases are considered to be false positives. The protocol had been transferred successfully to a second instrument maintaining the set action values, which correlated well with values established by the Public Health England (PHE) Sickle Cell & Thalassaemia screening programme [9]. Work had also been carried out to correlate observed ratios of the γ and β chain ratios to gestational age in premature samples and to age after birth. These ratios were used to guide interpretation of results obtained and to reduce the number of samples referred unnecessarily for second testing.

This work was overlapped by a multi-centre pilot study carried out by the PHE Sickle Cell & Thalassaemia screening programme during 2012 and 2013 [10,11]. The aim of the study was to investigate integration of the SpOtOn Clinical Diagnostics method into routine screening services, determine if common action values could be established for all manufacturers and laboratories, and assess consistency with existing methods. Four laboratories participated in the study; 23,878 samples were analysed using either ABI Sciex AP4000 (2 laboratories) or Waters Micromass (Xevo TQMS (1 laboratory) and Premier (1 laboratory) instruments. The study was unable to recommend the use of the latter instrument in this context, as false-positive rates were unacceptably high due to variable ratios. The need to replicate existing practice and ensure consistency with existing methods (HPLC, capillary electrophoresis (CE), and IEF) required the detection of carriers of the targeted haemoglobin variants as well as beta thalassaemia disease. Common action values were set for all experiments with the exception of Hb C, which has manufacturer-specific values [9].

The programme operates a two-test protocol such that results are only reported following second testing, so conservative action values were set to minimise the likelihood of false-negative results from the first line test. The action values are available online [9] and subject to ongoing review and optimisation with laboratories who have implemented the method. At the time of writing, three English newborn screening laboratories have implemented the protocol with a further three actively assessing the method. Over 150 positive cases have been identified in a cohort of approximately 250,000 babies.

The protocol has also been investigated for use in a German setting with 29,079 newborn samples screened as part of an evaluation project carried out between November 2015 and September 2016 in Berlin [12]. Samples were analysed in parallel with CE, with 100% concordance reported. Samples positive for sickle cell disease (*n* = 7) were also confirmed by molecular techniques. The authors have concluded that MSMS is a suitable technique for newborn screening in Germany, citing the benefits of the existing expertise in MSMS techniques as well as the ability to use software algorithms to only find sickle cell disease cases. This is in keeping with the requirements of their genetic testing act, which prohibits the testing of minors for heterozygous states considered to be not relevant in childhood or adolescence [12].

## 4. Summary

The development of ESI-MSMS for newborn sickle cell disease screening was driven by the concept that rationalization of resource within newborn screening laboratories would be of benefit in a health care environment where there is increasing pressure on equipment, cost, and workforce skill mix. The protocol uses the same equipment as that used for newborn metabolic screening. Initial set up is more time-consuming when compared to HPLC, and is similar to IEF, however analysis and result interpretation time is significantly reduced, particularly if software algorithms are used to scrutinize the data. This also has the advantage of removing operator-dependent variability. The method is targeted and more specific than existing procedures, whilst the inherent flexibility has enabled users to develop protocols that fit with local practice and requirements. Reported sensitivity for Hb S is 100%, where this data is presented [4,11,12]. Costs vary according to laboratory arrangements but are comparable with other available techniques.

Since the first report of the procedure in 2005 [3], the literature shows that the protocol has been adopted in a number of different settings, and it is estimated that more than one million babies have been screened with no false-negative cases reported. The ability to prevent the operator from identifying the majority of cases of carriers fulfils ethical requirements and is seen as advantageous by some users, although use of the protocol in this way does mean that some rare cases of sickle cell disease will be missed. The rationalization of equipment and skills, along with reduced interpretative requirements, is also advantageous in the current environment. Where screening programmes already exist, it is important to ensure standardization with existing practice, and this has been demonstrated to be possible in the English setting [10]. The current lack of an experiment to detect Hb Bart’s may limit uptake in areas where this currently falls into newborn screening requirements. However, strategies to target Hb Bart’s are being investigated by the manufacturer. Other areas under development include quality control material. 

In conclusion, ESI-MSMS has been shown to be a specific, sensitive, and practical technique for newborn screening for sickle cell disease.

## 5. Patents

WO2006082389A1, Screening method. WO2008/0135756A1, Peptide Standards.

## Figures and Tables

**Table 1:** Results of comparison of blood spots and the isoelectric focussing technique (IEF).

Haemoglobins Detected	Number (*n*)
Total samples tested	40,054
No abnormality detected	39,710
FS	9
FSC	3
FC	1
FAS	187
FAC	38
FAD^Punjab^	49
FAE	47
FAO^Arab^	0
Wild-type β absent ^#^	4

F = fetal; A = adult/wild type; ^#^ all subsequently confirmed as β thalassaemia disease.
